# Functional Transitions Among Older Adults in Rural China: Examining the Differential Roles of Care From Daughters’ and Sons’ Families

**DOI:** 10.1093/geronb/gbae133

**Published:** 2024-08-03

**Authors:** Pianpian Zhao, Yanan Zhang, Sarah Harper, Weihong Zeng, Shuzhuo Li

**Affiliations:** Jinhe Center for Economic Research, Xi’an Jiaotong University, Xi’an, Shaanxi, China; Oxford Institute of Population Ageing, University of Oxford, Oxford, UK; Oxford Institute of Population Ageing, University of Oxford, Oxford, UK; Jinhe Center for Economic Research, Xi’an Jiaotong University, Xi’an, Shaanxi, China; Center for Ageing and Health Research, School of Public Policy and Administration, Xi’an Jiaotong University, Xi’an, Shaanxi, China

**Keywords:** Care source, Daughter care advantage, Health inequality, Son preference

## Abstract

**Objectives:**

Informal care provided by adult children is of great importance for older adults’ well-being in China. This paper investigates and compares the functional transitions among older adults living in rural areas who receive care from daughters’ and from sons’ families.

**Methods:**

This study utilizes the “Well-being of Elderly Survey in Anhui Province” (WESAP) from 2001 to 2021. Our sample included 2,797 individuals aged 60 years or older. Functional status was based on the activities of daily living and the instrumental activities of daily living. We employed a random-effects ordered logit model to examine the functional transitions among the older adults.

**Results:**

Receiving care from daughters’ families is significantly associated with a lower likelihood of functional decline compared to receiving care from sons’ families in rural China. The advantage associated with daughter care becomes more pronounced among older individuals with a severe functional difficulty compared to those with a mild or moderate functional difficulty. The difference is prevalent among older adults aged 75 and older, with less wealth or multiple chronic diseases, or who live alone. Furthermore, among those with severe functional difficulties, the daughter advantage is more significant for fathers as compared to mothers.

**Discussion:**

Nowadays, daughters’ families can provide high-quality informal care, often surpassing that offered by sons’ families. This daughter advantage becomes even more significant among older adults who have a higher need for family care, such as those with severe disabilities and limited financial resources.

The prevalence of functional decline among older adults constitutes significant medical and societal challenges (Zhang et al., 2019), especially in countries like China where population aging is happening rapidly. The proportion of the Chinese population aged 65 and older has been consistently increasing, surging from 4.9% in 1982 to 14.9% in 2022 (NBSC, 2023), and is expected to reach approximately 30% by 2050 (Wang & Wang, 2021). China is witnessing an increase in the number of older adults living with disabilities. In 2020, this figure stood at an estimated 43.75 million, a number expected to double to nearly 90 million by 2050 (Zhang et al., 2020). These statistics position China as the country with the highest proportion of older adults living with disabilities worldwide (Tao & Zeng, 2022). Limitations in performing daily activities, a crucial stage in the progression toward disability, is seen as an inevitable part of aging process (Shur et al., 2021). It can ultimately lead to deteriorating quality of life, worsening health outcomes, and elevated morbidity rates (Fuchs et al., 2022).

The quality and nature of care that older adults receive significantly influence the trajectory of their functional decline (Berry et al., 2015; Holman, 2020). Family serves as the primary source to eldercare in China (Liu & Cook, 2018). Influenced by Confucian norms, China traditionally expects children to fully support their older parents (Harper & Zhang, 2023; Li, 2020). This responsibility typically falls to sons, who carry on the family lineage and inherit family assets in the patrilineal society (Hu, 2017). However, societal shifts over recent decades have seen changes to these norms. The reduction in family size, driven by the one-child policy, coupled with increasing female participation in the workforce, urbanization, and societal modernization, has gradually increased the need and acceptance of care from daughters (Zhang & Harper, 2022a). This shift has, in turn, highlighted concerns about potential differences in the care provided by sons and daughters, given the long-standing preference for sons and the pervasive gender gap in socioeconomic status.

Although prior research has explored the quantity and types of care given by daughters’ families and sons’ families (Gruijeters, 2017; Xie & Zhu, 2009), there are relatively few studies that examine the health outcomes resulting from this care (Zeng, Brasher, et al., 2016; Zhang & Harper, 2022a, 2022b). Existing literature suggests that care significantly influences the trajectory of older adults’ functional status (Read et al., 2021). However, little is known about how variations in care provided by different familial relationships affect the rate of functional decline in older adults.

To bridge this gap in the literature, our study contrasts the functional transitions of older parents cared for by their daughters’ families with those cared for by their sons’ families. This comparison is essential for understanding the complex interplay of gender roles, cultural expectations, and the impacts on both caregivers and care recipients. The findings will highlight the evolving roles of daughters and sons in patrilineal societies with reduced family sizes and demonstrate shifts in societal expectations and the distribution of caregiving responsibilities. Additionally, this study examines the impact of changing family dynamics on health inequality within contemporary China and assesses societal factors contributing to functional decline. This supports policymakers in early identification and intervention for those at risk of functional decline, facilitating the development of strategies to reduce health disparities, in accordance with WHO’s objectives for promoting healthy aging.

## Care and Functional Decline

The trajectory of functional decline in older adults is significantly shaped by the quality and nature of the care they receive (Read et al., 2021). High-quality family care is crucial for maintaining, and potentially improving, the abilities of older adults to perform activities of daily living (ADLs) and instrumental activities of daily living (IADLs). Compassionate and attentive caregiving contributes to a supportive atmosphere that reduces stress and physical strain for older adults. This nurturing atmosphere not only preserves their physical abilities but also promotes mental health by empowering them with decision-making in their care, thereby fostering a sense of autonomy and well-being (Berry et al., 2015).

Additionally, effective care proactively manages chronic conditions that might otherwise accelerate functional decline. This includes early symptom identification, timely intervention to prevent complications, and support for adherence to treatment and lifestyle adjustments aimed at mitigating the impact of chronic illnesses (Holman, 2020). Such regular, consistent care empowers older adults to maintain optimal health and quality of life.

Furthermore, family caregivers play a key role in introducing adaptive strategies and assistive devices that enhance safety and facilitate independence. The tailored support of family care is thus integral to prolonging an older adult’s ability to live independently. Providing a safe and comfortable living environment is fundamental to enhancing the life quality of older adults (Szanton et al., 2016). This environment promotes physical activity and encourages social interactions, both of which are essential for sustaining the physical and cognitive health of older adults (Stroke et al., 2023). Therefore, the high-quality care, which integrates physical and emotional support with health management and an engaging living space, may prevent functional decline and actively contribute to a life of independence and dignity.

## The Advantages in the Care From Daughters’ and Sons’ Families

Traditionally, eldercare in China has been the responsibility of sons, with daughters expected to care for their in-laws (Cong & Silverstein, 2012; Hu, 2017). However, with the implementation of the One-Child Policy and increased female labor participation, daughters are now more involved in providing both practical and financial support to their natal parents (Harper & Zhang, 2023). In this context, there is extensive research examining the amount and types of care provided by daughters’ and sons’ families (for a review of this topic and further discussions on the changes in family dynamics in China, please refer to Supplementary Material Section 1). However, the health outcomes associated with these care sources have received less attention (Liu & Harper, 2018; Zhang & Harper, 2022a).

A body of recent research suggests that older adults cared for by their sons or daughters-in-law report higher levels of self-perceived health and mental well-being, particularly in rural areas of China, compared to those who receive care from their daughters or sons-in-law (Cong & Silverstein, 2008; Liu & Harper, 2018; Zhang & Harper, 2022a, 2022b). Cultural norms in China expect that sons, more so than daughters, are obligated to provide care for their older parents. Additionally, the socioeconomic advantage often held by sons may enable them to secure better healthcare services and living conditions for their older parents. These factors have fostered a culture where sons are often seen as the preferred, and even superior, caregivers for older adults (Cong & Silverstein, 2012; Liu & Harper, 2018; Zhang & Harper, 2022b). When the source of care aligns with these cultural expectations, older adults are more likely to be satisfied and comfortable, thereby positively affecting their well-being (Cong & Silverstein, 2008).

Although son preference is prevalent, there are also advantages associated with daughter care. Gender ideologies often assert that daughters, due to their traditional roles in domestic work, are more adept at providing instrumental and emotional care compared to sons (Zeng, Brasher, et al., 2016). As caregiving tasks are predominately performed by women, eldercare from sons’ families is generally provided by daughters-in-law (Warmenhoven et al., 2018). Daughters typically maintain more intimate relationships with their natal parents than daughters-in-law do, making them more likely to better understand their parents’ needs and provide more considerate care (Shi, 2009). In addition, the frequently documented conflicts between parents-in-law and daughters-in-law could potentially reduce the well-being of co-residing older parents (Zeng, George, et al., 2016). Hence, care provided by daughters might offer certain advantages that enhance the well-being of older adults.

Several studies have discovered that parents often express greater satisfaction with care provided by daughters (Shi, 2009; Zeng, George, et al., 2016). Research using the Chinese Longitudinal Healthy Longevity Survey (CLHLS) between 2002 and 2009 even suggested that parents whose primary emotional caregiver was their daughter or son-in-law reported a higher level of cognitive capacity and a lower mortality rate compared to those who primarily received emotional care from their son or daughter-in-law (Zeng, George, et al., 2016). An analysis of three waves (2009, 2012, and 2015) of the WESAP longitudinal study found both mortality and objective morbidity risk were lower under daughter care, though subjective reported health status was better under son care (Liu & Harper, 2018).

## The Present Study

China presents notable disparities between rural and urban areas. In terms of economy, infrastructure, and social welfare system, rural areas are generally at a disadvantage (Cai & Yue, 2020; Zhang et al., 2015). For older adults residing in rural regions, accessibility to healthcare and formal social care services is limited (Wang et al., 2019). As a result, family care, especially the support provided by children, assumes a more pivotal role in these communities. Our study addresses the unexplored question of the relationship between the source of care and the rate of function decline among older adults in rural China. Our aim is to identify the factors associated with functional transition and health inequalities faced by the older population in these rural regions and inform strategies for effective intervention.

As aforementioned, in Chinese families, it is typically the daughters-in-law who provide care for older adults in sons’ families. The trend of urbanization over the past few decades has seen many younger individuals, particularly men, migrate from rural to urban areas in pursuit of better employment opportunities (Li et al., 2018). Often, they leave their families behind in the rural areas due to the high cost of living in urban areas. In such instances, some older adults are cared for by daughters-in-law in the absence of their sons. Given the closer bond between parents and their biological children, as compared to in-law relationships, and the traditional gender ideology that posits women as more skilled at caregiving, we anticipate that daughters may provide better care to their natal parents than both daughters-in-law and sons. Therefore, we hypothesize that parents who are cared for by daughters’ families may exhibit a slower rate of functional decline than those who receive care from sons’ families (Hypothesis 1).

This disparity may rely on the degree of older adults’ dependency on their children for care, influenced by a range of factors. For instance, those with severe functional difficulties may struggle with more daily tasks and self-care, requiring more intensive support from family caregivers. Therefore, their health is more susceptible to the quality of family care. Additionally, older adults with limited financial means may have no choice but to rely solely on unpaid care from their family. However, those with adequate financial resources have the option to seek professional care to supplement the insufficient family support, mitigating the influence of family care quality on their health. Moreover, advanced age typically brings a combination of these challenges, intensifying the dependency on family support. Building on this, we hypothesize that the difference in the functional decline in older adults receiving care from their sons’ and daughters’ families may be more significant among those who are more dependent on their children for care—such as those with severe functional difficulties, limited financial resources, who live alone, or who are of advanced age (Hypothesis 2).

Previous research (Zhang & Harper, 2022a, 2022b) found that mothers’ well-being is more dependent on their children’s filial piety and financial capacity compared to that of fathers. The gender ideology and the gender difference in life expectancy infer that fathers are more likely to receive companion and support from their partners, which lowers men’s dependency on their adult children. We thus expect that the differences in functional decline associated with the source of care may be more significant for mothers as compared to fathers (Hypothesis 3).

## Method

### Data and Sample

We used the 2001–2021 waves of Well-being of Elderly Survey in Anhui Province (WESAP), a longitudinal research project led jointly by the University of Southern California and the Institute for Population and Development Studies of Xi’an Jiaotong University in China (Li et al., 2009). The survey was established to better understand the well-being of the older adults in rural China. This data set covers a range of topics, including social and economic characteristics, family relations, intergenerational support, and physical and psychological well-being. The project, which employed a stratified, multistage, random-sampling methodology, was launched in 2001. It gathered data from individuals aged 60 years and older residing in the rural areas of the Chaohu in Anhui province, China. The second wave was collected in 2003 and then followed up at 3-year intervals (Zhang & Silverstein, 2022). Please see Supplementary Material Section 2 for further details about the region.

Our study began with a total sample of 11,577 participants from eight waves. As our investigation focuses on the sources of care, we excluded a portion of the sample (*n* = 6,074) that did not receive any informal care. Additionally, we excluded participants with no functional difficulty (*n* = 1,843), as this group may not need informal care. We also excluded those with missing follow-up measures (*n* = 484), as our analysis examines functional status changes across consecutive waves. Furthermore, we omitted participants with missing values for key variables (*n* = 379). Ultimately, our final sample encompassed 2,797 observations, drawn from 1,481 older adults. A comprehensive flowchart demonstrating our sample selection process can be found in Supplementary Material Section 3.

### Measurement

#### Transition in functional status

We apply six-item ADLs and nine-item IADLs to measure functional status. ADLs primarily involve basic self-care tasks essential for personal well-being and survival, such as bathing and eating. IADLs entail more complex activities necessary for maintaining one’s domestic life and sociocultural existence, such as shopping and cooking. For more information, please refer to Supplementary Material Section 4. Participants were asked if they could usually perform each of the six ADL or nine IADL tasks independently. They could respond in one of three ways: (1) on your own, (2) with assistance from someone else, or (3) not at all. If the participant responded with either 2 or 3, they were categorized as having difficulty performing that task.

Drawing from established literature (Jagger et al., 2001; Lopez & Nicodemo, 2016), we defined five levels of functional status:

(1) No functional difficulty: The participant can perform all six ADLs and nine IADLs independently.
(2) Mild functional difficulty: The participant can perform all six ADLs independently but experiences difficulty with at least one IADL.
(3) Moderate functional difficulty: The participant experiences difficulty performing 1–2 ADLs.
(4) Severe functional difficulty: The participant experiences difficulty performing 3 or more ADLs.
(5) Death: The participant passed away.

We created an ordinal three-category variable, *FuncTrans*, to document changes in respondents’ functional status across the two consecutive waves. The categories are: (1) transition to a lower level of functional difficulty, (2) no change in functional difficulty, and (3) transition to a severer level of functional difficulty.

#### Care source

The survey collected information about who assists the respondents with household tasks (such as cleaning, laundry, and cooking) and ADLs (like bathing, toileting, and eating). Respondents could report multiple caregivers if they received assistance from more than one person. We classified the care recipients (*n* = 2,797) according to their relationship with their caregivers:

(1) Son family care: Respondents who received care from at least one son or daughter-in-law but not from a daughter or son-in-law (*n* = 913).
(2) Daughter family care: Respondents who received care from at least one daughter or son-in-law, but not from a son or daughter-in-law (*n* = 460).
(3) Both care: Respondents who received care from both sons’ and daughters’ families (*n* = 391).
(4) Other care: Respondents who received care exclusively from individuals other than their children (*n* = 1,033).

Supplementary Table 1 provides an overview of the caregiving structure. Note that older adults in groups 1–3 may receive care from others in addition to their children, such as spouses and siblings. We created four binary variables, namely, *SonFamilyCare*, *DaughterFamilyCare*, *BothCare*, and *OtherCare*, to represent each group, respectively.

### Baseline Models

To compare the functional transition among older parents who receive care from different family, we set up the baseline model as follows:


$$\begin{array}{llll} FuncTrans_{i,t}= & \;\;\beta_{1}SonFamilyCare_{i,t}+\beta_{2}DaughterFamilyCare_{i,t}\; & +\;\beta_{3}BothCare_{i,t}+\beta_{4}Controls_{i,t}\; & +u_{i}+u_{t}+u_{i,t} \end{array}$$ (1)

where $FuncTrans_{i,t}$ represents the changes in level of functional status from *t* to *t* + *1* for individual *i*. This is an ordinal categorical variable taking on three distinct values: “1” for functional improvement, “2” for no change in functional status, and “3” for functional decline. *SonFamilyCare*_*i,t*_ is a binary variable that is set to one if the respondent *i* receives care from their sons’ families but not their daughters’ families in year *t*, and zero otherwise. *DaughterFamilyCare*_*i,t*_ and *BothCare*_*i,t*_ follow the same binary assignment for care received from the daughters’ families and from both sons’ and daughters’ families, respectively. Respondents receiving care from sources other than their children, such as from spouses, serve as the reference group.

*Controls*_*i,t*_ comprises a set of variables identified as significant determinants influencing older adults’ functional well-being. They include age, gender, marital status, education level, income, number of children, self-rated health, and life satisfaction (Chen et al., 2014; Connolly et al., 2016). Detailed definitions and measurements of all these variables can be found in Supplementary Table 2. In the model, *u*_*i*_ denotes individual-specific effects, capturing unobserved individual characteristics that are constant over time. *u*_*t*_ represents the business cycle effects, accounting for time-variant macroeconomic conditions that may influence the functional well-being of all individuals. Lastly, *u*_*i,t*_ symbolizes the idiosyncratic error term, capturing any unmeasured influences on the functional status change for individual *i* at time *t* that are not accounted for by the other terms in the model.

In the initial model, it’s possible that a respondent who receives care from their sons’ (daughters’) family, rather than their daughters’ (sons’), might also have support from other individuals, such as a spouse or siblings. To exclude the potential effects of care from other sources, we conducted a robustness check by directly comparing the functional outcomes of older adults who exclusively receive care from either their sons’ families or their daughters’ families with the following model:


$$\begin{array}{lll} FuncTrans_{i,t}\;= & \;\beta_{1}OnlyDaughterFamilyCare_{i,t}\; & +\;\beta_{2}Controls_{i,t}+u_{i}+u_{t}+u_{i,t} \end{array}$$ (2)

Where *OnlyDaughterFamilyCare*_*i,t*_ is a binary variable that equals 1 if the respondent solely receives care from the daughters’ families and 0 otherwise. The reference group consists of respondents who receive care exclusively from their sons’ families.

### Estimation Methodology

Because the dependent variable *FuncTrans* is an ordinal outcome variable and the intervals between the three categories are not equal, we adopt an ordered logistic regression model to examine the likelihood of an older adult falling into a certain category (Fullerton, 2009). In addition, to control for unobserved heterogeneity or individual-specific effects, we apply the ordered logit model with random effect. Further details regarding the choice of the random-effects model are provided in Supplementary Material Section 5. Cluster-robust standard errors are applied to adjust the calculation of standard errors to account for possible correlation of errors within individuals as well as heteroskedasticity (MacKinnon et al., 2022). All the analyses have been conducted with Stata 15.

## Results

### The Transition in Functional Status

**Figure 1.**
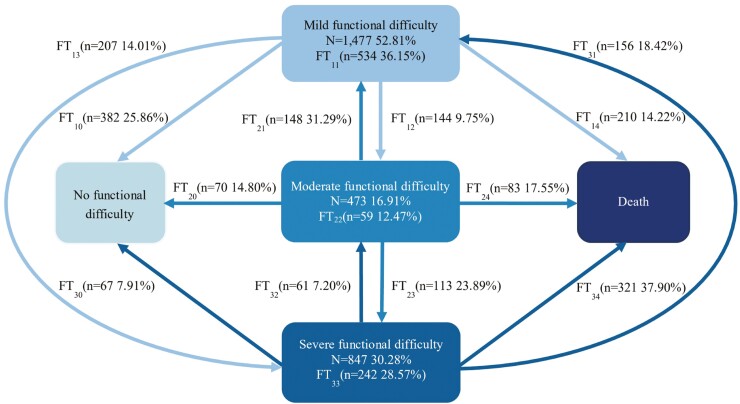
Transition in functional status among older adults in rural China. There are five functional statuses: “0” for no functional difficulty, “1” for mild functional difficulty, “2” for moderate functional difficulty, “3” for severe functional difficulty, and “4” for death. *FT*_*i,j*_ represents transitions from function status *i* to *j*, and arrows indicate those transitions.

Figure 1 displays 2,797 episodes of transitions in functional status. Among the 1,477 transitions that originated from a state of mild functional difficulty, 534 (36.15%) of these individuals maintained their initial functional level, whereas 382 (25.86%) showed functional improvements and moved into a state of no functional difficulty. Conversely, 144 (9.75%) of these respondents shifted to moderate functional difficulty, 207 (14.01%) transitioned into severe functional difficulty, and 210 (14.22%) passed away.

For transitions commencing from a moderate functional difficulty level, out of 473 cases, 59 (12.47%) remained stable in their functional status. Meanwhile, 218 (46.09%) exhibited functional improvements, with 70 (14.80%) transitioning to no functional difficulty and 148 (31.29%) transitioning to mild functional difficulty. In contrast, 113 (23.89%) escalated to severe disability, and 83 (17.55%) individuals passed away. Finally, among the 847 cases transitioning from severe disability, 242 (28.57%) remained static in their functional status, whereas 321 (37.90%) individuals passed away. Meanwhile, 284 (33.53%) demonstrated episodes of functional improvement: 67 (7.91%) transitioned to a state with no functional difficulty, 156 (18.42%) eased into mild functional difficulty, and 61 (7.20%) improved to moderate functional difficulty.

### Descriptive Statistics

Table 1 illustrates the characteristics of our pooled sample. Within the 2,797 respondents, 1,764 (63.07%) are recipients of care from their children. Of these, 913 (32.64%) are taken care of by their sons’ families, 460 (16.45%) receive care from their daughters’ families, and 391 (13.98%) have the support of both their sons’ and daughters’ families. The respondents have an average age of 75.037 years, with 55.8% of them being married, and approximately 22.6% have completed primary education.

**Table 1.** Descriptive Statistics of the Variables

	Full sample	Care providers			
		Sons’ families	Daughters’ families	Both	Other
	*N* = 2,797	*N* = 913	*N* = 460	*N* = 391	*N* = 1,033
Variables	*M* (*SD*) or %	*M* (*SD*) or %	*M* (*SD*) or %	*M* (*SD*) or %	*M* (*SD*) or %
Age	75.037 (7.754)	77.573 (7.576)	74.372^***^ (7.42)	75.885 (7.891)	72.771 (7.270)
Gender					
Female	0.617	0.720	0.641^**^	0.668	0.498
Male	0.383	0.280	0.359^**^	0.332	0.502
Marital status					
Married	0.558	0.315	0.480^***^	0.435	0.855
Widowed	0.427	0.679	0.511^***^	0.565	0.113
Divorced	0.005	0.005	0.004	—	0.006
Never married	0.010	—	0.004	—	0.026
Educational level					
Illiterate	0.774	0.851	0.826	0.780	0.680
Primary education	0.226	0.149	0.174	0.220	0.320
Middle school	0.033	0.018	0.022	0.026	0.054
High school	0.005	0.004	0.002	0.003	0.009
Income	2,408.907 (3,347.508)	1,907.678 (3,013.303)	2,565.019^***^ (3,878.187)	2,188.725 (3,263.099)	2,865.734 (3,340.962)
Logarithm of income	6.938 (1.884)	6.647 (1.955)	6.981^**^ (1.817)	6.775 (1.977)	7.238 (1.766)
Number of children	3.691 (1.668)	3.724 (1.605)	3.741 (1.69)	4.169 (1.378)	3.459 (1.770)
Self-rated health					
SRH_Good	0.136	0.170	0.146	0.113	0.111
SRH_Fair	0.425	0.427	0.411	0.442	0.424
SRH_Poor	0.438	0.403	0.443	0.445	0.465
Life satisfaction	12.501 (2.378)	12.852 (2.307)	12.293^***^ (2.433)	12.632 (2.364)	12.233 (2.382)
Transition in functional status, *N* (%)					
Improvement	884 (31.61)	218 (23.88)	160^***^ (34.78)	106 (27.11)	400 (38.72)
No change	835 (29.85)	299 (32.75)	130 (28.26)	112 (28.64)	294 (28.46)
Decline	1,078 (38.54)	396 (43.37)	170^**^ (36.96)	173 (44.25)	339 (32.82)

*Notes*: We have conducted mean-comparison tests between the care provider groups of sons’ families and daughters’ families. *SD* = standard deviation; SRH = self-rated health.

^*^ *p* < .05.

^**^ *p* < .01.

^***^ *p* < .001.

Respondents cared for by their daughters’ families tend to be younger (74.372 vs 77.573 years), male (35.9% vs 28.0%), and married (48.0% vs 31.5%) and have a higher income (6.981 vs 6.647) than those cared for by their sons’ families. Additionally, they display a lower life satisfaction score (12.293 vs 12.852) and are less prone to experience a functional decline as compared to their counterparts who receive care from their sons’ families (36.96% vs 43.37%).

### Transition in Functional Status and Source of Care

Table 2 presents the random-effects ordered logit estimates examining the relationship between functional status transitions and care sources. Column 1 reveals that, for respondents receiving care from their sons’ families, the odds ratio of experiencing a functional decline (versus improving their functional status or maintaining the same level) is 1.256 times that of those cared for by other (non-child) sources (95% CI [1.035, 1.523]). Contrastingly, the odds ratio for older adults receiving care from their daughters’ families does not significantly differ from those receiving care from non-child sources (OR = 1.082, 95% CI [0.862, 1.359]). Column 2 indicates that the odds ratio for older parents exclusively receiving care from their daughters’ families is 0.667 times (0.496, 0.898) that of participants exclusively cared for by their sons’ families, and this difference is statistically significant.

**Table 2.** Random-Effects Ordered Logistic Regression Models for Functional Transitions and Care Provided by Sons’ and Daughters’ Families

Variables	Functional transitions	
	(1)	(2)
Care source		
Model 1 (Ref: OtherCare)		
SonFamilyCare	1.256^*^ [1.035, 1.523]	
DaughterFamilyCare	1.082 [0.862, 1.359]	
BothCare	1.320^*^ [1.029, 1.693]	
Model 2 (Ref: OnlySonFamilyCare)		
OnlyDaughterFamilyCare		0.667^**^ [0.496, 0.898]
Age	1.092^***^ [1.079, 1.105]	1.098^***^ [1.073, 1.123]
Gender (Ref: Women)		
Men	1.054 [0.895, 1.240]	1.045 [0.741, 1.473]
Marital status (Ref: Widowed, divorced, and never married)		
Married	1.144 [0.959, 1.364]	1.313 [0.895, 1.925]
Education (Ref: Illiterate)		
Primary education	0.889 [0.730, 1.084]	0.856 [0.529, 1.385]
Income	0.951 [0.905, 1.001]	1.030 [0.934, 1.136]
Number of children	0.988 [0.948, 1.030]	0.951 [0.878, 1.030]
Self-rated health (SRH; Ref: SRH_Good and SRH_Fair)		
SRH_Poor	1.257^**^ [1.073, 1.472]	1.075 [0.790, 1.461]
Life satisfaction	0.975 [0.944, 1.007]	0.967 [0.908, 1.029]
Log pseudolikelihood	−2,831.5	−833.1
Chi-squared	423.4	127.9
Number of group	1,481	576
*N*	2,797	844

*Notes:* Odds ratios are reported, and the 95% confidence intervals are in brackets. Year dummies are included in all models, but their coefficients are not reported for brevity. See Supplementary Material for the complete definitions of all variables.

^*^ *p* < .05.

^**^ *p* < .01.

^***^ *p* < .001.

### Transition in Functional Status and Source of Care by Initial Functional Status

Panel A in Table 3 presents the random effects ordered logit estimates, detailing the relationship between transitions in functional status and the source of care, grouped by the initial functional status. Columns 1–2, which focus on participants with mild, and moderate functional difficulty, indicate no significant difference in the odds ratio of functional decline (as opposed to functional improvement or maintaining the same level of functionality) for parents who receive care from their sons’ (1.216, [0.908, 1.629]; 1.037, [0.617, 1.742]) or daughters’ (1.287, [0.920, 1.802]; 1.369, [0.767, 2.443]) families, when compared to those receiving care from others.

**Table 3.** Random-Effects Ordered Logistic Regression Models for Functional Transitions and Care Provided by Sons’ and Daughters’ Families by Initial Functional Status

Variables	(1)	(2)	(3)
	From mild functional difficulty	From moderate functional difficulty	From severe functional difficulty
Panel A: Model 1			
Care source (Ref: OtherCare)			
SonFamilyCare	1.216 [0.908, 1.629]	1.037 [0.617, 1.742]	1.770^**^ [1.174, 2.669]
DaughterFamilyCare	1.287 [0.920, 1.802]	1.369 [0.767, 2.443]	0.852 [0.543, 1.337]
BothCare	1.354 [0.922, 1.988]	1.678 [0.908, 3.099]	1.620^*^ [1.007, 2.607]
Log pseudolikelihood	−1,404.5	−428.9	−840.8
Chi-squared	177.5	65.1	64.8
Number of group	1,008	406	614
*N*	1,477	473	847
Panel B: Model 2			
Care source (Ref: OnlySonFamilyCare)			
OnlyDaughterFamilyCare	0.753 [0.461, 1.231]	1.368 [0.542, 3.453]	0.273^***^ [0.152, 0.489]
Log pseudolikelihood	−381.6	−112.0	−257.3
Chi-squared	57.2	16.3	54.1
Number of group	354	122	220
*N*	446	132	266

*Notes:* Odds ratios are reported, and the 95% confidence intervals are in brackets. Age, gender, marital status, education level, income, number of children, self-rated health, life satisfaction, and year dummies are included in all models, but their coefficients are not reported for brevity. The complete form is available upon request. See Supplementary Material for the complete definitions of all variables.

^*^ *p* < .05.

^**^ *p* < .01.

^***^ *p* < .001.

However, when considering older adults with severe functional difficulty, as shown in Column 3 of Panel A, the odds ratio for those under the care of their sons’ families is 1.770 times (1.174, 2.669) that of those receiving care from other sources. There is no significant difference in the odds ratios between individuals receiving care from their daughters’ families and those cared for by others (0.852, [0.543, 1.337]).

Panel B restricts the sample to those solely cared for by their daughters’ or sons’ families. Columns 1–2 exhibit no significant association between functional transitions and care sources for individuals with mild (0.753, [0.461, 1.231]) or moderate (1.368, [0.542, 3.453]) functional difficulty. However, for those with severe functional difficulty, the odds ratio for older adults solely cared for by their daughters’ families is 0.273 times (0.152, 0.489) that of those solely cared for by their sons’ families (Column 3).

### Heterogeneities Among Demographic and Socioeconomic Groups

Table 4 discloses the relationship between transition in functional status and care source across various demographic and socioeconomic characteristics, focusing on those with severe functional difficulty. We have also conducted the same analyses for the full sample and those experiencing mild or moderate functional difficulties. These results are presented in Supplementary Table 3. In line with the findings in Table 3, the significant association between the odds ratio of functional decline and care source is observed only in the group with severe functional difficulties. For brevity, this section will specifically concentrate on the results for this group.

**Table 4.** Heterogeneities Across Demographic and Socioeconomic Groups: Older Adults With Severe Functional Difficulties

Variables	Gender		Age		Wealth		Chronic diseases		Live alone	
	(1)	(2)	(3)	(4)	(5)	(6)	(7)	(8)	(9)	(10)
	Women	Men	≤75	>75	Top 25%	Bottom 75%	≤1	>1	No	Yes
Care source (Ref: OtherCare)										
SonFamilyCare	1.657^*^ [1.019, 2.693]	2.250^*^ [1.122, 4.511]	2.148 [0.857, 5.382]	1.821^*^ [1.115, 2.974]	0.955 [0.376, 2.428]	2.194^***^ [1.424, 3.382]	1.224 [0.524, 2.859]	1.976^*^ [1.145, 3.411]	1.295 [0.641, 2.616]	2.279^*^ [1.207, 4.304]
DaughterFamilyCare	1.057 [0.606, 1.843]	0.523 [0.231, 1.182]	0.522 [0.231, 1.178]	1.128 [0.616, 2.068]	1.010 [0.412, 2.480]	0.819 [0.493, 1.361]	1.090 [0.346, 3.441]	0.890 [0.525, 1.510]	0.823 [0.362, 1.874]	0.809 [0.447, 1.464]
BothCare	1.305 [0.742, 2.296]	2.628^*^ [1.113, 6.206]	1.448 [0.540, 3.881]	1.888^*^ [1.056, 3.376]	1.624 [0.623, 4.238]	1.764^*^ [1.047, 2.973]	1.898 [0.729, 4.944]	1.593 [0.885, 2.867]	1.353 [0.619, 2.958]	1.692 [0.841, 3.403]
Log pseudolikelihood	−533.6	−291.2	−298.4	−531.1	−155.5	−674.8	−197.7	−626.5	−392.8	−433.6
Chi-squared	105.0	37.8	27.5	60.8	45.79	104.0	37.3	40.6	77.0	49.2
Number of group	378	243	264	385	155	498	180	488	311	347
*N*	539	308	318	529	173	674	208	639	398	443

*Notes:* We use a random-effects ordered logit model. Odds ratios are reported, and the 95% confidence intervals are in brackets. Age, gender, marital status, education level, income, number of children, self-rated health, life satisfaction, and year dummies are included in all models, but their coefficients are not reported for brevity. The complete form is available upon request. See Supplementary Material for the complete definitions of all variables.

^*^ *p* < .05.

^**^ *p* < .01.

^***^ *p* < .001.

Grouped by gender, Column 1 shows that mothers receiving care from their sons’ families exhibit an odds ratio for functional decline (versus either functional improvement or maintenance) at 1.657 times (1.019, 2.693) that of mothers cared for by non-child sources. For fathers, as presented in Column 2, the odds ratio of respondents cared for by their sons’ families is 2.250 times (1.122, 4.511) that of those receiving care from non-child sources. There is no significant difference in the odds ratio between those cared for by their daughters’ families and those supported by non-child sources among both mothers and fathers.

The association between the odds ratio of functional declines and the care source is observed in both mothers and fathers. To further explore this disparity, we have conducted a supplementary analysis that introduces an interaction between the care recipient’s gender and the care source. This approach enables a direct comparison of these associations between fathers and mothers, with the findings detailed in Supplementary Table 4. The analysis consistently reveals that mothers who receive care exclusively from daughters’ families are associated with a lower odds ratio of functional decline compared to those receiving care solely from sons’ families (0.413 [0.229, 0.744]). Among fathers, this difference is significantly more pronounced (0.207 [0.050, 0.848]), indicating a notably larger association when compared to mothers.

The source of care is not significantly associated with functional transitions among younger older adults (Column 3), participants in the top 25% income distribution (Column 5), individuals with at most one chronic disease (Column 7), and those not living alone (Column 9). In contrast, participants aged 75 and older who receive care from their sons’ families show higher odds ratio of functional decline compared to those cared for by non-child sources (Column 4: 1.821 [1.115, 2.974]). Similar trends are seen for older adults with lower income levels (Column 6: 2.194 [1.424, 3.382]), those with multiple chronic diseases (Column 8: 1.976 [1.145, 3.411]), and those living alone (Column 10: 2.279 [1.207, 4.304]). However, among these groups, there’s no statistically significant difference in the odds ratios between those receiving care from their daughters’ families and those cared for by non-child sources.

### Sensitivity Analysis

To ensure the robustness of our results, we perform several sensitivity analyses, and the results and discussions are provided in Supplementary Material Section 7.

## Discussion and Conclusion

This study expands upon the limited knowledge surrounding the transition in functional status among older adults cared for by their sons’ and daughters’ families in rural China, using eight waves of WESAP. We find a lower likelihood of functional decline in older adults receiving care from their daughters’ families compared to those receiving care from their sons’ families. This “daughter advantage” in caregiving is more pronounced among fathers and those more dependent on their children.

The better health outcomes resulting from care provided by daughters’ families, as opposed to sons’ families (Hypothesis 1), aligns with findings from Zeng, Brasher, et al. (2016), Zeng, George, et al. (2016), and Shi (2009). This can be attributed to the prevailing gender ideology that suggests women are more adept at caregiving than men, reinforcing the caregiving role of daughters. Furthermore, in sons’ families, the responsibility of caring for older family members often falls to the daughters-in-law (Youn et al., 1999). However, since parents usually share a stronger emotional bond with their biological children than with their in-laws, daughters might be more attuned to the needs of their aging parents. This understanding can facilitate more timely and effective caregiving (Shi, 2009). As a result, such care could potentially lead to a slower deterioration in the older parents’ functional abilities.

This finding contradicts the deep-rooted cultural norm and the gender gap in socioeconomic status suggesting sons as the optimal caregivers for the older parents as noted in the earlier research (Zhang & Harper, 2022b). The shift might be attributable to factors such as modernization and increased labor force participation among women, both of which bolster women’s empowerment. This empowerment enables women to negotiate family responsibilities with their parents-in-law and partners and to instrumentally and financially support their natal parents (Shi, 2009; Yan, 2016). Moreover, the enactment of the one-child policy has led to a reduction in family sizes and a decrease in the number of available sons, thereby increasing both the acceptance and need for daughters to provide care (Zhang & Harper, 2022a). All these factors make care from daughters’ families an increasingly appealing and effective option for eldercare over time.

The literature that supports the son advantage, such as Zhang & Harper (2022a, 2022b), employs self-rated health status and mental well-being, both of which are subjectively based. In contrast, our study uses ADL and IADL measurements, and Zeng, George, et al. (2016) rely on mortality rate and cognitive decline, all of which disclose a daughter advantage. This discrepancy supports earlier work that disclose the source of care influences subjective and objective health outcomes differently (Liu & Harper, 2018). Care provided by sons may have more favorable psychological outcomes, as it follows the traditional cultural norms. However, in practice, daughters might offer superior practical care, thus improving objective health outcomes. Our Table 1 echoes with this point, disclosing that older parents cared for by their daughters’ families report a lower level of functional decline and a lower level of life satisfaction. This hypothesis warrants further investigation.

The results also emphasize that the disparity in functional transition between older parents cared for by daughters’ family versus sons’ families is primarily observed in groups that have a high demand for family care (Hypothesis 2). These groups include individuals with severe functional difficulties, those aged 75 and older, older adults with limited resources, respondents with multiple chronic diseases, and those who live alone. Compared to sons and daughters-in-law, daughters may often display greater patience and empathy toward their aging parents (Shi, 2009). This heightened sensitivity becomes especially vital for these particularly vulnerable groups, which can potentially slow down functional decline and enhance overall health outcomes.

However, our findings do not support our original hypothesis that “daughter advantage” in caregiving is more pronounced among mothers compared to fathers (Hypothesis 3). In the context of caregiving, some tasks necessitate a certain level of physical intimacy, such as bathing and personal hygiene assistance. It has been observed that older adults typically feel more at ease when receiving such personal care from a child of the same gender (Wong, 2005) or from an intimate relationship (McPherson, 2011). However, due to the predominant role of daughters and daughters-in-law as family caregivers, older men often lack the option for same-gender care. In some cases, fathers might conceal their need for more intimate forms of assistance from their daughters-in-law, which could adversely affect their functional status. Consequently, older fathers may express a stronger preference for care from daughters as opposed to daughters-in-law; a preference that could potentially be more significant than in the case of older mothers. This may lead to a more significant caregiving difference in terms of functional transitions favoring daughters among fathers, especially among those with severe functional difficulties.

### Limitations

This study is not without its limitations. First, the causal interpretation of our study relies heavily on the strict exogeneity assumption concerning the source of care. However, there may be endogeneity caused by omitted variables that simultaneously influence both the source of care and the functional decline of older adults. These omitted variables could be due to data limitations or the difficulty in quantifying factors such as the willingness, motivation, or capability of children to provide care. Despite this, the association found between the functional decline among older adults and the care provided by the families of daughters and sons can assist us in identifying groups whose health is at disadvantage.

Additionally, our analysis focuses on a specific rural area in China, characterized by a large outflow of young people and a high proportion of older adults. There are also more developed rural areas in China with greater work opportunities and a more diverse demographic mix, such as Huaxi in Jiangsu Province. Therefore, our results may not be universally applicable to all rural areas in China. However, our study using WESAP specifically targets the typical rural regions, making our sample particularly suitable for this topic. Moreover, our measurement of functional decline is based on changes observed across two survey waves, which were collected 3 years apart. However, significant changes in functional status could occur within those 3 years that our study design would not capture. The data would only reflect the status at the beginning and end of the 3-year period, potentially missing shorter-term fluctuations or gradual changes that happen within that time frame. This is also a common problem in most studies examining the changes in health status (Chen et al., 2014; van Houwelingen et al., 2013). Future research should seek to use more comprehensive measurement of changes in functional status when additional data become available, to provide a more nuanced understanding of the dynamics of functional decline.

### Implications

As the Chinese population continues to age, deepening our understanding of the progression of functional decline (seen as a natural aspect of the aging process), is essential for the establishment of preventative measures and the preparation of future health and social care services. Using the research data on rural older adults with one of the longest survey durations, our study enriches this understanding by revealing the social determinants of functional decline and highlighting the important role of the source of care in rural China. Traditionally, sons were the primary caregivers and inheritors in China, but societal shifts have increasingly led daughters to take on the role of caring for their older parents. Our study also sheds light on the evolving dynamics of the care system in China by exploring the difference in care provided by daughters’ families and sons’ families.

Our findings indicate a health disparity among older adults with different sources of care. Older adults without access to care from daughters may face a higher risk of functional decline, highlighting their vulnerability and the need for support. This contradicts the cultural expectation that sons are the superior caregivers for older adults and underscores the necessity to reevaluate social norms regarding the roles of daughters and sons. A reduction in household size, resulting in limited availability of family care, emphasizes the increasingly critical role that community services and policy interventions must play to bridge this gap. This necessitates the development of strategies that guarantee the provision of suitable, high-quality care to all older adults, regardless of their family dynamics or living arrangements. Additionally, our findings highlight that fathers with severe difficulties are more affected by the source of care compared to mothers, a trend potentially attributable to the absence of same-gender care. Hence, it seems imperative to encourage and train more professional male caregivers or involve sons more actively in caregiving responsibilities.

Our study provides important implications for future research. Family dynamics in modern China are constantly evolving, with women’s labor participation rates steadily increasing. With reduced family sizes and increased work responsibilities among women, critical questions arise. These include how care is managed and whether sons and sons-in-law will become more involved in caregiving, especially when there are simultaneous care demands from multiple family members, such as both parents-in-law and natal parents. Examining these dynamics and their effects on the well-being of both caregivers and recipients is essential for future studies, incorporating both qualitative and quantitative research methods.

In summary, our study underscores the crucial role of the source of care in functional transitions, finding a daughter advantage in providing eldercare. It also indicates a need to reassess societal norms and expectations around caregiving roles, particularly in traditional societies with a strong son preference. Furthermore, to address this health inequality raised from care source, it is imperative to design and implement a comprehensive long-term care system. Such a system should aim to provide equitable care and promote the health and well-being of our aging population.

## Supplementary Material

gbae133_suppl_Supplementary_Materials

## Data Availability

For access to the Well-being of Elderly Survey in Anhui Province, please direct inquiries to the Institute for Population and Development Studies, School of Public Policy and Administration, Xi’an Jiaotong University, Xi’an, Shaanxi, China (ipds@mail.xjtu.edu.cn). For access to the STATA codes used in the analyses, please contact the corresponding author. The studies reported in this manuscript were not preregistered.
