# Supplementary material for: Functional Transitions Among Older Adults in Rural China: Examining the Differential Roles of Care From Daughters’ and Sons’ Families
Source: J Gerontol B Psychol Sci Soc Sci. 2024 Aug 3;79(10):gbae133. doi: 10.1093/geronb/gbae133 (PMC11447736; doi:10.1093/geronb/gbae133)
Supplement: gbae133_suppl_Supplementary_Materials [file gbae133_suppl_supplementary_materials.docx]

***The Journals of Gerontology, Series B: Psychological Sciences and Social Sciences* Supplementary Material: Zhao et al. Functional Transitions among Older Adults in Rural China: Examining the Differential Roles of Care from Daughters’ and Sons’ Families.**

**Section 1: The role of child in eldercare in China**

Deeply influenced by Confucian norms and further compounded by an underdeveloped social welfare system, older adults in China primarily rely on their families, particularly their children, for various forms of support (Harper & Zhang, 2023). This eldercare responsibility is further complicated by China’s traditionally patrilineal society, where sons typically carry on the family lineage and inherit most of the family assets (Hu, 2017). In turn, sons and their spouses are tasked with providing care for their ageing parents (Cong & Silverstein, 2012). Conversely, daughters, following marriage, become a part of their husband’s family and are expected to take care of their in-laws (Hu, 2017). Historically in China, men assumed the role of breadwinners, while women primarily dedicated themselves to household duties and family care, attending to both their own nuclear family and their in-laws. This deep commitment not only limited their availability but also constrained their participation in the labour force. Consequently, parents typically did not anticipate receiving either financial or physical support from their married daughters (Shi, 2009).

Over recent decades, Chinese society has increasingly accepted the care provided by daughters for their older parents (Zhang & Harper, 2022a). The implementation of the One-Child Policy from 1979 to 2015 has dramatically reduced household size in China, affecting the availability of family care for older adults (Lei, 2013) and increasing the necessity and demand for daughters' involvement. Additionally, women's increased labour participation along with the economic development, has improved their financial capacity. Coupled with the advancement of gender equality during the modernization process, these factors have collectively empowered women, enhancing their ability and responsibility to provide both practical and financial support to their birth parents (Yan, 2016; Shi, 2009).

Types of support are generally classified into instrumental, financial, and emotional categories. The majority of findings suggest that sons are more likely to provide and offer a larger amount of financial support to their parents (Zeng et al., 2016a). Conversely, while there is less literature on emotional support, it consistently reveals that daughters are more likely to offer emotional support to their natal parents (Gruijeters, 2017). Regarding instrumental support, the results are more mixed, but they reveal an emerging trend where daughters play an increasingly significant role in taking care of their natal parents (Zhan & Montgomery, 2003; Sam, 2022). This trend is particularly pronounced in urban areas where sons’ availability is lower and modernization and economic development are more advanced.

**Section 2: Region description**

Anhui, a predominantly rural province, is one of the China's most populous regions, with 61.13 million people by the end of 2021 (Xie et al., 2022). The province has high proportion of older adults and a substantial out-migration of its working-age population (Zhang & Silverstein, 2022). Chaohu was selected for the survey as a typical rural area, located in the heart of Anhui with a population of approximately 4.5 million (Xu et al., 2020). The area is conveniently located a short train journey away from Hefei, Nanjing, and Shanghai — cities with abundant employment opportunities (Du, 2015), and many local residents work or migrate to these cities.

**Section 3: Sample selection**

**Supplementary Figure 1.** *Screening process*

**Section 4: ADLs and IADLs**

ADLs primarily involve basic self-care tasks essential for personal well-being and survival. In the survey, these are represented by six activities: bathing, eating, dressing, getting out of bed, walking across a room, and using the toilet. Meanwhile, IADLs entail more complex activities necessary for maintaining one's domestic life and sociocultural existence. The survey measures IADL with nine tasks: lifting 10 kg, climbing a flight of stairs, bending, squatting, or kneeling, walking about 100 meters, cooking, shopping, cycling independently, doing housework, and managing money.

**Section 5: Random-effects model**

In panel data analysis, a fixed-effects model is beneficial for controlling unobserved characteristics that remain constant over time. However, in our analysis, the main independent variables, which represent the care structure, show limited variation over time within our sample. For example, among those with severe functional difficulties who are cared for by their daughters, less than 15% experienced changes in their care source. This accounts for a relatively small proportion of the observations. Under these conditions, random-effects estimators prove to be more efficient, particularly considering that our sample size is not large (Wooldridge, 2010). These estimators operate under the assumption that individual-specific effects are not correlated with the independent variables. The random-effects ordered logit model has been widely applied in many areas of research, including social science and biomedical studies (Hedeker & Gibbons, 1994).

**Section 6: Supplementary Tables**

**Supplementary Table 1.** *Care structure*

| **Participants who were experiencing functional transitions and receiving care (n=2,797)** | | | |
| --- | --- | --- | --- |
| Participants received care from their child  (n=1,764 63.07%) | Care from son’s family  (n=913 32.64%) | Care from son’s family only  (n=597 21.34%) | Only son (n=130 4.65%) |
|  |  |  | Only daughter-in-law (n=223 7.97%) |
|  |  |  | Son and daughter-in-law (n=244 8.72%) |
|  |  | Care from son’s family and spouse (n=175 6.26%) | |
|  |  | Care from son’s family and other (n=120 4.29%) | |
|  |  | Care from son’s family, spouse and other (n=21 0.75%) | |
|  | Care from son’s family and daughter’s family  (n=391 13.98%) | Care from son’s family and daughter’s family (n=209 7.47%) | |
|  |  | Care from son’s family, daughter’s family and spouse (n=94 3.36%) | |
|  |  | Care from son’s family, daughter’s family and other (n=64 2.29%) | |
|  |  | Care from son’s family, daughter’s family, spouse and other (n=24 0.86%) | |
|  | Care from daughter’s family  (n=460 16.45%) | Care from daughter’s family only  (n=247 8.83%) | Only daughter (n=216 7.72%) |
|  |  |  | Only son-in-law (n=4 0.14%) |
|  |  |  | Daughter and son-in-law (n=27 0.97%) |
|  |  | Care from daughter’s family and spouse (n=144 5.15%) | |
|  |  | Care from daughter’s family and other (n=49 1.75%) | |
|  |  | Care from daughter’s family, spouse and other (n=20 0.72%) | |
| Participants didn't receive care from child (n= 1033 36.93%) | Care from spouse (n=820 29.32%) | | |
|  | Care from spouse and other (n=171 6.11%) | | |
|  | Care from other (n=42 1.50%) | | |

**Supplementary Table 2.** *Definitions of Variables*

| **Variable** | **Definition** |
| --- | --- |
| **Age** | Respondent’s age |
| **Gender** |  |
| **Women** | Equal to 1 if the respondent is female, 0 otherwise |
| **Men** | Equal to 1 if the respondent is male, 0 otherwise |
| **Marital status** |  |
| **Married** | Equal to 1 if the respondent is married, 0 otherwise |
| **Widowed** | Equal to 1 if the respondent is widowed, 0 otherwise |
| **Divorced** | Equal to 1 if the respondent is divorced, 0 otherwise |
| **Never married** | Equal to 1 if the respondent is never married, 0 otherwise |
| **Educational level** |  |
| **Illiterate** | Equal to 1 if the respondent is illiterate, 0 otherwise |
| **Primary Education** | Equal to 1 if the respondent has completed primary education, 0 otherwise |
| **Middle School** | Equal to 1 if the respondent has completed middle school education, 0 otherwise |
| **High School** | Equal to 1 if the respondent has completed high school, 0 otherwise |
| **Income^a^** | Respondents' total annual net household income |
| **Logarithm of income** | Logarithm of respondent's total annual net household income |
| **Number of children** | The number of respondent’s children |
| **Self-rated health** |  |
| **SRH_Good** | Equal to 1 if the respondent reports good health, 0 otherwise |
| **SRH_Fair** | Equal to 1 if the respondent reports fair health, 0 otherwise |
| **SRH_Poor** | Equal to 1 if the respondent reports bad health, 0 otherwise |
| **Life satisfaction^b^** | Life satisfaction score of respondents, ranging from 0 to 8. |
| **Care source** |  |
| **SonFamilyCare** | Equal to 1 if the respondent received care from sons’ families, 0 otherwise |
| **DaughterFamilyCare** | Equal to 1 if the respondent received care from daughters’ families, 0 otherwise |
| **BothCare** | Equal to 1 if the respondent received care from sons’ families and daughters families, 0 otherwise |
| **OtherCare** | Equal to 1 if the respondent received care from others rather than children, 0 otherwise |
| **OnlySonFamilyCare** | Equal to 1 if the respondent received care from sons’ families only, 0 otherwise |
| **OnlyDaughterFamilyCare** | Equal to 1 if the respondent received care from daughters’ families only, 0 otherwise |
| **OnlyCareFromDaughter-in-law** | Equal to 1 if the respondent received care from daughter-in-law only, 0 otherwise |
| **OnlyCareFromDaughter** | Equal to 1 if the respondent received care from daughter only, 0 otherwise |
| **Functional status** | Category of functional status: 1 No functional difficulties; 2 Mild functional difficulties; 3 Moderate functional difficulties; 4 Severe functional difficulties; 5 Death |
| **Functional transition** | Functional transition of the respondents: 1 Improvement; 2 No change; 3 Decline |
| **Improvement** | Equal to 1 if the respondent transitions to a lower level of functional difficulty, 0 otherwise |
| **No change** | Equal to 1 if the respondent's level of functional difficulty remains constant, 0 otherwise |
| **Decline** | Equal to 1 if the respondent transitions to a higher level of functional difficulty, 0 otherwise |

*Note.* a Respondents' total annual net household income, including net income from employment, pensions and other sources in the past year. b Life satisfaction is measured by eight questions: "Do you think your life is better than other people's?", "Are you satisfied with your life?", “Do you find it is interesting to do what you do?”, “Are these years the best years in your life?”, “Do you want to change your past life if possible?”, “Do you find most of the things you do are boring?”, “Do you think you are old and life is not interesting?”, and “Do you think most of your life is in line with your expectation”. The higher the score, the more satisfied the respondent is.

**Supplementary Table 3.** Heterogeneities across demographic and socioeconomic groups

| **Variables** | **Gender** | | **Age** | | **Wealth** | | **Chronic disease** | | **Live alone** | |
| --- | --- | --- | --- | --- | --- | --- | --- | --- | --- | --- |
|  | **(1)** | **(2)** | **(3)** | **(4)** | **(5)** | **(6)** | **(7)** | **(8)** | **(9)** | **(10)** |
|  | **Women** | **Men** | **≤75** | **>75** | **Top 25%** | **Bottom 75%** | **≤1** | **>1** | **No** | **Yes** |
| **Panel A: Full sample** | | | | | | | | | | |
| **Care source (Ref: OtherCare)** |  |  |  |  |  |  |  |  |  |  |
| **SonFamilyCare** | 1.144 | 1.416^*^ | 1.086 | 1.474^*^ | 1.092 | 1.313^*^ | 1.248 | 1.235 | 1.277 | 1.21 |
|  | [0.888,1.476] | [1.034,1.939] | [0.841,1.402] | [1.097,1.982] | [0.739,1.613] | [1.038,1.661] | [0.873,1.783] | [0.976,1.563] | [0.938,1.737] | [0.925,1.585] |
| **DaughterFamilyCare** | 1.086 | 1.013 | 1.016 | 1.183 | 1.304 | 1.014 | 1.463 | 0.982 | 1.133 | 1.027 |
|  | [0.804,1.467] | [0.698,1.469] | [0.750,1.375] | [0.828,1.691] | [0.866,1.962] | [0.775,1.326] | [0.954,2.244] | [0.751,1.283] | [0.755,1.698] | [0.777,1.358] |
| **BothCare** | 1.113 | 1.735^**^ | 1.208 | 1.511^*^ | 1.505 | 1.283 | 1.274 | 1.315 | 1.348 | 1.260 |
|  | [0.810,1.529] | [1.141,2.638] | [0.867,1.683] | [1.034,2.209] | [0.954,2.373] | [0.947,1.738] | [0.801,2.027] | [0.989,1.748] | [0.922,1.969] | [0.889,1.785] |
| **Log pseudolikelihood** | -1748.6 | -1050.4 | -1447.9 | -1371.8 | -686.1 | -2130.8 | -889.0 | -1924.0 | -1263.3 | -1537.3 |
| **Chi-squared** | 269.7 | 165.7 | 128.8 | 98.2 | 130.7 | 246.3 | 170.6 | 254.7 | 244.9 | 185.9 |
| **Number of Group** | 861 | 655 | 928 | 786 | 550 | 1190 | 709 | 1128 | 813 | 924 |
| ***N*** | 1727 | 1070 | 1406 | 1391 | 704 | 2093 | 920 | 1877 | 1284 | 1491 |
| **Panel B: Subgroup with mild functional difficulties** | | | | | | | | | | |
| **Care source (Ref: OtherCare)** |  |  |  |  |  |  |  |  |  |  |
| **SonFamilyCare** | 1.263 | 1.135 | 1.091 | 1.424 | 0.968 | 1.304 | 1.625 | 1.020 | 1.436 | 1.052 |
|  | [0.837,1.905] | [0.742,1.736] | [0.775,1.535] | [0.870,2.330] | [0.568,1.651] | [0.923,1.844] | [0.926,2.852] | [0.721,1.444] | [0.928,2.223] | [0.708,1.565] |
| **DaughterFamilyCare** | 1.283 | 1.357 | 1.378 | 1.157 | 1.708 | 1.186 | 1.818^*^ | 1.072 | 1.396 | 1.220 |
|  | [0.798,2.063] | [0.813,2.265] | [0.938,2.024] | [0.641,2.088] | [0.993,2.939] | [0.800,1.759] | [1.015,3.256] | [0.726,1.582] | [0.783,2.490] | [0.823,1.807] |
| **BothCare** | 1.265 | 1.514 | 1.131 | 1.724 | 1.351 | 1.366 | 1.695 | 1.212 | 1.705 | 1.160 |
|  | [0.760,2.107] | [0.818,2.802] | [0.720,1.775] | [0.917,3.241] | [0.703,2.597] | [0.870,2.146] | [0.865,3.322] | [0.782,1.876] | [0.950,3.060] | [0.721,1.866] |
| **Log pseudolikelihood** | -823.5 | -551.6 | -857.7 | -538.5 | -385.7 | -1007.8 | -533.2 | -856.2 | -605.8 | -782.3 |
| **Chi-squared** | 125.0 | 123.6 | 134.5 | 74.4 | 90.0 | 135.0 | 48.0 | 197.4 | 192.8 | 153.0 |
| **Number of Group** | 588 | 431 | 660 | 470 | 358 | 774 | 497 | 666 | 506 | 614 |
| ***N*** | 905 | 572 | 854 | 623 | 405 | 1072 | 583 | 894 | 670 | 800 |
| **Panel C: Subgroup with moderate functional difficulties** | | | | | | | | | | |
| **Care source (Ref: OtherCare)** |  |  |  |  |  |  |  |  |  |  |
| **SonFamilyCare** | 0.715 | 1.795 | 0.973 | 0.882 | 2.674 | 0.828 | 0.687 | 1.257 | 1.171 | 0.607 |
|  | [0.353,1.447] | [0.716,4.498] | [0.147,6.433] | [0.420,1.851] | [0.757,9.449] | [0.457,1.503] | [0.228,2.069] | [0.681,2.322] | [0.478,2.868] | [0.277,1.330] |
| **DaughterFamilyCare** | 1.193 | 1.763 | 1.541 | 1.081 | 1.708 | 1.294 | 0.904 | 1.513 | 1.635 | 1.127 |
|  | [0.530,2.684] | [0.734,4.230] | [0.592,4.011] | [0.422,2.770] | [0.507,5.751] | [0.649,2.581] | [0.214,3.827] | [0.785,2.914] | [0.568,4.706] | [0.569,2.232] |
| **BothCare** | 1.318 | 2.192 | 3.852 | 0.796 | 4.239^*^ | 1.236 | 0.553 | 2.344^*^ | 1.807 | 1.136 |
|  | [0.586,2.967] | [0.736,6.530] | [0.078,190.841] | [0.294,2.155] | [1.292,13.906] | [0.571,2.677] | [0.120,2.558] | [1.179,4.660] | [0.687,4.751] | [0.445,2.897] |
| **Log pseudolikelihood** | -256.4 | -164.1 | -206.6 | -208.9 | -106.5 | -310.9 | -99.2 | -315.6 | -185.3 | -227.0 |
| **Chi-squared** | 42.6 | 28.6 | 7.7 | 39.4 | 32.8 | 38.2 | 846.1 | 51.3 | 41.2 | 35.6 |
| **Number of Group** | 241 | 169 | 214 | 208 | 111 | 311 | 124 | 303 | 195 | 216 |
| ***N*** | 283 | 190 | 234 | 239 | 126 | 347 | 129 | 344 | 216 | 248 |

*Note.* We use a random-effects ordered logit model. Odds ratios are reported, and the 95% confidence intervals are in brackets. Age, gender, marital status, education level, income, number of children, self-rated health, life satisfaction, and year dummies are included in all models, but their coefficients are not reported for brevity. The complete form is available upon request. See Supplementary Material for the complete definitions of all variables.

**p* <.05, ***p* <.01, ****p* <.001.

**Supplementary Table 4.** *Interaction between gender and care source*

| **Variables** | **(1)** |
| --- | --- |
|  | **Functional transitions** |
| **Care source (Ref: OnlySonFamilyCare)** |  |
| **OnlyDaughterFamilyCare** | 0.413^**^ |
|  | [0.229, 0.744] |
| **Men # OnlyDaughterFamilyCare** | 0.207^*^ |
|  | [0.050, 0.848] |
| **Age** | 1.102^***^ |
|  | [1.058, 1.148] |
| **Gender (Ref: Women)** |  |
| **Men** | 2.019 |
|  | [0.922, 4.420] |
| **Marital status (Ref: Widowed, divorced and never married)** | |
| **Married** | 2.000 |
|  | [0.826, 4.844] |
| **Education (Ref: Illiterate)** |  |
| **Primary Education** | 0.633 |
|  | [0.264, 1.516] |
| **Income** | 0.952 |
|  | [0.849, 1.069] |
| **Number of children** | 0.829^*^ |
|  | [0.716, 0.960] |
| **Self-rated health (Ref: SRH_Good and SRH_Fair)** |  |
| **SRH_poor** | 1.476 |
|  | [0.866, 2.516] |
| **Life satisfaction** | 0.970 |
|  | [0.871,1.081] |
| **Log pseudolikelihood** | -259.0 |
| **Chi-squared** | 49.3 |
| **Number of Groups** | 220 |
| ***N*** | 266 |

*Note.* We use a random-effects ordered logit model. Odds ratios are reported, and the 95% confidence intervals are in brackets. See Supplementary Material for the complete definitions of all variables.

**p* <.05, ***p* <.01, ****p* <.001.

**Section 7: Sensitivity analysis**

Given that daughters-in-law are often the primary caregivers for the older adults in sons’ families, we have narrowed our sample to include older adults who receive care exclusively from daughters or daughters-in-law as a robustness check. They are detailed in Supplementary Table 5. It demonstrates that the odds ratio of functional decline for parents exclusively cared for by their daughters is 0.621 times (0.419, 0.920) that of those solely cared for by their daughters-in-law.

We also utilize two alternate measures - dummy variables indicating functional decline and functional improvement (Supplementary Table 6). Furthermore, to address the prevalence of missing data primarily found in *Functional Transition* and *Life Satisfaction*, we utilized the Multiple Imputation by Chained Equations (MICE) technique. We implemented ordered logit and linear regression models that contained all variables from the initial model for *Functional Transition* and *Life Satisfaction* respectively. We conducted this procedure over 20 imputation cycles. The results can be found in Supplementary Tables 7 and 8. These additional analyses align with our primary findings, reinforcing the validity and reliability of our results.

**Supplementary Table 5.** *Random-effects ordered* *logistic regression models for functional transitions and care provided by daughter and daughter-in-law*

| **Variables** | **Functional transitions** |
| --- | --- |
|  | **OR**  **[95% CI]** |
| **Care source (Ref: OnlyCareFromDaughter-in-law)** |  |
| **OnlyCareFromDaughter** | 0.621* |
|  | [0.419, 0.920] |
| **Age** | 1.083*** |
|  | [1.050, 1.117] |
| **Gender (Ref: Women)** |  |
| **Men** | 0.687 |
|  | [0.424, 1.113] |
| **Marital status (Ref: Widowed, divorced and never married)** |  |
| **Married** | 1.229 |
|  | [0.745, 2.027] |
| **Education (Ref: Illiterate)** |  |
| **Primary Education** | 0.961 |
|  | [0.472, 1.957] |
| **Income** | 1.073 |
|  | [0.917, 1.254] |
| **Number of children** | 0.916 |
|  | [0.812, 1.032] |
| **Self-rated health (Ref: SRH_Good and SRH_Fair)** |  |
| **SRH_Poor** | 0.813 |
|  | [0.537, 1.230] |
| **Life satisfaction** | 0.935 |
|  | [0.857, 1.021] |
| **Log pseudolikelihood** | -438.5 |
| **Chi-squared** | 66.1 |
| **Number of Group** | 342 |
| ***N*** | 439 |

*Note.* Odds ratios are reported, and the 95% confidence intervals are in brackets. Year dummies are included in all models, but their coefficients are not reported for brevity. See Supplementary Material for the complete definitions of all variables.

**p* <.05, ***p* <.01, ****p* <.001.

**Supplementary Table 6.** Sensitivity analysis of functional transitions and care provided by son's and daughter's family: with new dependent variables

| **Variables** | **Functional decline** | | **Functional improvement** | |
| --- | --- | --- | --- | --- |
|  | **(1)** | **(2)** | **(3)** | **(4)** |
|  | **Model 1** | **Model 2** | **Model 1** | **Model 2** |
|  | **OR**  **[95% CI]** | **OR**  **[95% CI]** | **OR**  **[95% CI]** | **OR**  **[95% CI]** |
| **Care Source** |  |  |  |  |
| **Model 1 (Ref: OtherCare)** |  |  |  |  |
| **SonFamilyCare** | 1.898^**^ |  | 0.583^*^ |  |
|  | [1.218,2.959] |  | [0.355,0.958] |  |
| **DaughterFamilyCare** | 1.109 |  | 1.500 |  |
|  | [0.659,1.867] |  | [0.901,2.497] |  |
| **BothCare** | 1.944^*^ |  | 0.726 |  |
|  | [1.167,3.241] |  | [0.410,1.287] |  |
| **Model 2 (Ref: OnlySonFamilyCare)** |  |  |  |  |
| **OnlyDaughterFamilyCare** |  | 0.261^**^ |  | 4.228^*^ |
|  |  | [0.111,0.616] |  | [1.022,17.502] |
| **Age** | 1.097^***^ | 1.102^**^ | 0.906^***^ | 0.902^*^ |
|  | [1.063,1.132] | [1.034,1.173] | [0.879,0.934] | [0.823,0.989] |
| **Gender (Ref: Women)** |  |  |  |  |
| **Men** | 2.358^***^ | 2.124 | 0.648^*^ | 1.623 |
|  | [1.553,3.582] | [0.969,4.656] | [0.425,0.990] | [0.725,3.634] |
| **Marital status (Ref: Widowed, divorced and never married)** |  |  |  |  |
| **Married** | 1.421 | 1.893 | 1.015 | 0.674 |
|  | [0.939,2.150] | [0.690,5.192] | [0.647,1.591] | [0.217,2.097] |
| **Education (Ref: Illiterate)** |  |  |  |  |
| **Primary Education** | 0.700 | 0.649 | 1.010 | 1.636 |
|  | [0.440,1.115] | [0.238,1.766] | [0.641,1.593] | [0.495,5.408] |
| **Income** | 0.933 | 1.052 | 1.124 | 1.085 |
|  | [0.837,1.040] | [0.857,1.292] | [0.991,1.273] | [0.838,1.405] |
| **Number of children** | 0.896^*^ | 0.757^**^ | 1.078 | 1.202 |
|  | [0.809,0.993] | [0.618,0.927] | [0.968,1.201] | [0.954,1.515] |
| **Self-rated health (Ref: SRH_Good and SRH_Fair)** |  |  |  |  |
| **SRH_Poor** | 1.674^**^ | 1.578 | 0.464^***^ | 0.733 |
|  | [1.168,2.400] | [0.796,3.130] | [0.314,0.686] | [0.323,1.662] |
| **Life satisfaction** | 0.951 | 1.037 | 1.074 | 1.110 |
|  | [0.890,1.017] | [0.909,1.183] | [0.997,1.157] | [0.928,1.326] |
| **Log pseudolikelihood** | -499.3 | -153.4 | -467.4 | -134.0 |
| **Chi-squared** | 51.6 | 19.0 | 72.1 | 8.8 |
| **Number of Groups** | 614 | 220 | 614 | 220 |
| ***N*** | 847 | 266 | 847 | 266 |

*Note*. The sample is among older parents with severe functional difficulties. We use a random effect logit model. Odds ratios are reported, and the 95% confidence intervals are in brackets. Year dummies are included in all models, but their coefficients are not reported for brevity. See Supplementary Material for the complete definitions of all variables.

**p* <.05, ***p* <.01, ****p* <.001.

**Supplementary Table 7**. Sensitivity analysis of functional transitions and care provided by son's and daughter's family: with imputations

| **Variables** | **Functional decline** | | **Functional improvement** | |
| --- | --- | --- | --- | --- |
|  | **(1)** | **(2)** | **(3)** | **(4)** |
|  | **Model 1** | **Model 2** | **Model 1** | **Model 2** |
| **Care source** |  |  |  |  |
| **Model 1 (Ref: OtherCare)** |  |  |  |  |
| **SonFamilyCare** | 0.626*** |  | 0.554** |  |
|  | [0.305, 0.947] |  | [0.379, 0.811] |  |
| **DaughterFamilyCare** | 0.185 |  | 0.987 |  |
|  | [-0.219, 0.589] |  | [0.646, 1.509] |  |
| **BothCare** | 0.667*** |  | 0.565* |  |
|  | [0.273, 1.062] |  | [0.348, 0.915] |  |
| **Model 2 (Ref: OnlySonFamilyCare)** |  |  |  |  |
| **OnlyDaughterFamilyCare** |  | 0.412** |  | 2.168* |
|  |  | [0.216, 0.784] |  | [1.064, 4.418] |
| **Age** | 0.092*** | 1.096*** | 0.909*** | 0.912** |
|  | [0.068, 0.116] | [1.040, 1.154] | [0.886, 0.933] | [0.858, 0.970] |
| **Gender (Ref: Women)** |  |  |  |  |
| **Men** | 0.701*** | 1.826 | 0.718 | 1.421 |
|  | [0.397, 1.004] | [0.991, 3.367] | [0.504, 1.025] | [0.704, 2.870] |
| **Marital status**  **(Ref: Widowed, divorced and never married)** | |  |  |  |
| **Married** | 0.355* | 1.877 | 0.810 | 0.734 |
|  | [0.044, 0.666] | [0.805, 4.377] | [0.567, 1.156] | [0.284, 1.894] |
| **Education (Ref: Illiterate)** |  |  |  |  |
| **Primary Education** | -0.197 | 0.671 | 0.984 | 1.382 |
|  | [-0.551, 0.157] | [0.294, 1.531] | [0.660, 1.466] | [0.590, 3.234] |
| **Income** | -0.034 | 1.070 | 1.124* | 1.061 |
|  | [-0.124, 0.056] | [0.899, 1.273] | [1.011, 1.250] | [0.868, 1.296] |
| **Number of children** | -0.057 | 0.829* | 1.060 | 1.137 |
|  | [-0.135, 0.020] | [0.707, 0.972] | [0.973, 1.155] | [0.963, 1.341] |
| **Self-rated health (Ref: SRH_Good and SRH_Fair)** |  |  |  |  |
| **SRH_Poor** | 0.268* | 1.491 | 0.572*** | 0.702 |
|  | [0.002, 0.535] | [0.842, 2.641] | [0.422, 0.775] | [0.359, 1.372] |
| **Life satisfaction** | -0.032 | 1.023 | 1.034 | 1.065 |
|  | [-0.088, 0.024] | [0.917, 1.142] | [0.973, 1.100] | [0.932, 1.218] |
| **Constant** | -7.904*** | 0.000** | 386.063*** | 67.140 |
|  | [-10.101, -5.707] | [0.000, 0.044] | [38.815, 3,839.867] | [0.659, 6,837.962] |
| **Number of groups** | 920 | 299 | 920 | 299 |
| ***N*** | 1,394 | 369 | 1,394 | 369 |

*Note*. The sample is among older parents with severe functional difficulties. We use a random effect logit model. Odds ratios are reported, and the 95% confidence intervals are in brackets. Year dummies are included in all models, but their coefficients are not reported for brevity. See Supplementary Material for the complete definitions of all variables.

**p* <.05, ***p* <.01, ****p* <.001.

**Supplementary Table 8.** Sensitivity analysis of functional declines and care provided by son's and daughter's family by demographic and socioeconomic characteristics: with imputations

| **Variables** | **Gender** | | **Age** | | **Wealth** | | **Chronic disease** | | **Live alone** | |
| --- | --- | --- | --- | --- | --- | --- | --- | --- | --- | --- |
|  | **(1)** | **(2)** | **(3)** | **(4)** | **(5)** | **(6)** | **(7)** | **(8)** | **(9)** | **(10)** |
|  | **Women** | **Men** | **<=75** | **>75** | **Top 25%** | **Bottom 75%** | **<=1** | **>1** | **No** | **Yes** |
| **Care source (Ref: OtherCare)** |  |  |  |  |  |  |  |  |  |  |
| **SonFamilyCare** | 1.679* | 2.340** | 1.366 | 2.118*** | 1.331 | 1.985*** | 1.367 | 2.089*** | 1.763* | 1.986** |
|  | [1.099, 2.565] | [1.349, 4.060] | [0.599, 3.113] | [1.461, 3.070] | [0.496, 3.571] | [1.401, 2.812] | [0.657, 2.843] | [1.371, 3.181] | [1.002, 3.104] | [1.206, 3.271] |
| **DaughterFamilyCare** | 1.471 | 0.856 | 1.075 | 1.267 | 1.331 | 1.196 | 0.977 | 1.346 | 1.324 | 1.067 |
|  | [0.877, 2.466] | [0.431, 1.701] | [0.508, 2.278] | [0.770, 2.083] | [0.404, 4.387] | [0.771, 1.854] | [0.339, 2.820] | [0.847, 2.137] | [0.623, 2.814] | [0.631, 1.805] |
| **BothCare** | 1.454 | 3.639*** | 1.926 | 2.144** | 1.526 | 2.028** | 1.690 | 2.096** | 1.879* | 2.184* |
|  | [0.871, 2.426] | [1.820, 7.279] | [0.717, 5.176] | [1.336, 3.441] | [0.488, 4.767] | [1.299, 3.166] | [0.670, 4.262] | [1.276, 3.444] | [1.007, 3.507] | [1.190, 4.009] |
| **Age** | 1.115*** | 1.088*** | 1.074 | 1.117*** | 1.102* | 1.096*** | 1.101** | 1.099*** | 1.106*** | 1.094*** |
|  | [1.062 1.169] | [1.052, 1.125] | [0.980, 1.177] | [1.071, 1.166] | [1.009, 1.203] | [1.066, 1.127] | [1.031, 1.175] | [1.057, 1.143] | [1.060, 1.155] | [1.054, 1.136] |
| **Gender (Ref: Women)** |  |  |  |  |  |  |  |  |  |  |
| **Men** |  |  | 1.987 | 2.006*** | 1.982 | 2.014*** | 1.841 | 2.144*** | 2.104** | 1.974** |
|  |  |  | [0.816, 4.842] | [1.368, 2.941] | [0.830, 4.736] | [1.443, 2.810] | [0.875, 3.873] | [1.465, 3.138] | [1.253, 3.532] | [1.309, 2.977] |
| **Marital status (Ref: Widowed, divorced and never married)** | | | | | | | | | | |
| **Married** | 1.785* | 1.136 | 1.369 | 1.429 | 1.397 | 1.445* | 0.660 | 1.901*** | 1.508 | 1.372 |
|  | [1.146, 2.781] | [0.699, 1.848] | [0.731, 2.562] | [0.972, 2.102] | [0.488, 4.000] | [1.031, 2.026] | [0.306, 1.426] | [1.321, 2.737] | [0.872, 2.610] | [0.908, 2.072] |
| **Education (Ref: Illiterate)** |  |  |  |  |  |  |  |  |  |  |
| **Primary Education** | 0.766 | 0.849 | 0.940 | 0.799 | 0.634 | 0.860 | 1.308 | 0.715 | 0.804 | 0.893 |
|  | [0.390, 1.504] | [0.541, 1.331] | [0.522, 1.695] | [0.498, 1.282] | [0.261, 1.540] | [0.579, 1.276] | [0.558, 3.068] | [0.469, 1.090] | [0.454, 1.425] | [0.555, 1.436] |
| **Income** | 0.936 | 1.003 | 0.815 | 1.038 | 1.024 | 0.941 | 1.106 | 0.924 | 0.991 | 0.966 |
|  | [0.830, 1.056] | [0.862, 1.167] | [0.579, 1.147] | [0.919, 1.172] | [0.377, 2.782] | [0.845, 1.047] | [0.894, 1.368] | [0.829, 1.030] | [0.857, 1.147] | [0.856, 1.090] |
| **Number of children** | 0.964 | 0.942 | 0.988 | 0.919 | 0.998 | 0.932 | 0.949 | 0.953 | 0.905 | 0.969 |
|  | [0.872, 1.067] | [0.829, 1.071] | [0.832, 1.173] | [0.841, 1.005] | [0.781, 1.275] | [0.858, 1.013] | [0.797, 1.130] | [0.865, 1.049] | [0.790, 1.037] | [0.884, 1.063] |
| **Self-rated health (Ref: SRH_Good and SRH_Fair)** | | | | | | | | | | |
| **SRH_poor** | 1.213 | 1.569 | 1.978 | 1.179 | 2.958 | 1.193 | 1.838 | 1.296 | 1.428 | 1.315 |
|  | [0.851, 1.729] | [0.976, 2.522] | [0.964, 4.058] | [0.852, 1.631] | [0.979, 8.941] | [0.896, 1.588] | [0.961, 3.513] | [0.924, 1.819] | [0.938, 2.172] | [0.896, 1.931] |
| **Life satisfaction** | 0.962 | 0.962 | 1.032 | 0.948 | 0.935 | 0.966 | 1.021 | 0.951 | 0.965 | 0.955 |
|  | [0.891, 1.039] | [0.872, 1.061] | [0.901, 1.181] | [0.888, 1.013] | [0.773, 1.130] | [0.909, 1.027] | [0.903, 1.155] | [0.888, 1.018] | [0.880, 1.058] | [0.885, 1.030] |
| **Constant** | 0.000*** | 0.001*** | 0.002 | 0.000*** | 0.000 | 0.001*** | 0.000** | 0.000*** | 0.000*** | 0.000*** |
|  | [0.000, 0.008] | [0.000, 0.025] | [0.002, 0.002] | [0.000, 0.003] | [0.000, 0.000] | [0.000, 0.007] | [0.000, 0.041] | [0.000, 0.012] | [0.000, 0.008] | [0.000, 0.012] |
| **Number of groups** | 563 | 374 | 372 | 616 | 180 | 798 | 297 | 739 | 453 | 556 |
| ***N*** | 889 | 505 | 474 | 920 | 203 | 1,191 | 347 | 1,047 | 616 | 766 |

*Note*. The sample is among older parents with severe functional difficulties. The dependent variable is Functional Decline. We use a random effect logit model. Odds ratios are reported, and the 95% confidence intervals are in brackets. Year dummies are included in all models, but their coefficients are not reported for brevity. See Supplementary Material for the complete definitions of all variables.

**p* <.05, ***p* <.01, ****p* <.001.

**References**

Du, H. (2015). *Leaving home from Chaohu: patterns and meanings of migration of educated young people* [PhD Thesis]. <https://research-information.bris.ac.uk/en/publications/leaving-home-from-chaohu-patterns-and-meanings-of-migration-of-ed>

Hedeker, D., & Gibbons, R. D. (1994). A Random-Effects Ordinal Regression Model for Multilevel Analysis. *Biometrics*, *50*(4), 933–944. <https://doi.org/10.2307/2533433>

Lei, L. (2013). Sons, Daughters, and Intergenerational Support in China. *Chinese Sociological Review*, *45*(3), 26–52. <https://doi.org/10.2753/csa2162-0555450302>

Sam, I. (2022). From bringing up sons to raising daughters for old age: Patrilineal beliefs regarding old-age security in the Chinese mainland. *Chinese Journal of Sociology*, *8*(4), 474–498. <https://doi.org/10.1177/2057150x221129338>

Wooldridge, J. M. (2010). Econometric analysis of cross section and panel data. MIT press.

Xie, H., Xu, T., Wu, Q., Zhang, M., Tong, N., & Zhang, T. (2022). Spatial and Economic Effects of Yangtze River-Huaihe River Water Transfer Project on the Transportation Accessibility of Bulk Cargo within Anhui Province, China. *Sustainability*, *14*(12), 7029. <https://doi.org/10.3390/su14127029>

Xu, J., Wu, Z., Schimmele, C. M., & Li, S. (2020). Widowhood and depression: a longitudinal study of older persons in rural China. *Aging & Mental Health*, *22*(6), 914–922. <https://doi.org/10.1080/13607863.2019.1571016>

Zhan, H. J., & Montgomery, R. J. V. (2003). Gender And Eldercare In China. *Gender & Society*, *17*(2), 209–229. <https://doi.org/10.1177/0891243202250734>
